# The Effect of Growth and Nutrition in Black Soldier Fly Larvae Fed by Hemp Seed Oil Mixed Diets

**DOI:** 10.3390/insects16111081

**Published:** 2025-10-23

**Authors:** Suttida Suwannayod, Phattawin Setthaya, Kwankamol Limsopatham, Napat Harnpornchai

**Affiliations:** 1 International College Digital Innovation, Chiang Mai University, Chiang Mai 50200, Thailand; suttida.su@cmu.ac.th; 2 Division of Product Development Technology, Faculty of Agro-Industry, Chiang Mai University, Chiang Mai 50200, Thailand; phatthawin.l@cmu.ac.th; 3 Department of Parasitology, Faculty of Medicine, Chiang Mai University, Chiang Mai 50200, Thailand; kwankamol.l@cmu.ac.th; 4 Faculty of Economics, Chiang Mai University, Chiang Mai 50200, Thailand

**Keywords:** *Hermetia illucens*, hemp seed oil, black soldier fly larvae (BSFL), sustainable animal feed

## Abstract

The black soldier fly (BSF), *Hermetia illucens,* has gained increasing attention as a sustainable protein source for animal feed. This study investigated the effects of dietary supplementation with hemp seed oil (HSO) at 0.5–6% concentrations on the growth performance and nutritional composition of black soldier fly larvae (BSFL). Larval development, survival rate, body weight, and adult longevity were evaluated under controlled conditions. In addition, chemical characterization of HSO was performed, and the proximate composition, mineral content, and amino acid profile of dried larvae were analyzed. The results indicated that HSO supplementation had no statistically significant effect on developmental time, survival rate, biomass accumulation, or adult lifespan compared to the control. Gas Chromatography–Mass Spectrometry (GC-MS) profiling of HSO revealed a wide range of bioactive compounds, including unsaturated fatty acids (UFAs), phytosterols, cannabinoids, and tocopherols. The BSFL showed high levels of fat and energy, with essential amino acids and minerals present in favorable concentrations for feed applications. The HSO improves the protein levels at the 0.5–1.0% concentration, with negative correlations at higher concentrations. The findings suggest that HSO can be integrated into BSFL diets without adverse effects on growth performance, while potentially enhancing the functional value of the larvae. This supports the feasibility of incorporating hemp by-products into insect-rearing systems to promote circular and value-added feed production.

## Simple Summary

Black soldier fly larvae (BSFL) are being studied as a new source of protein for animals, as they can turn food waste into valuable nutrients. This study explores whether adding hemp seed oil (HSO) to the BSFL diet causes no harm to the larvae and no negative impact on their growth or survival. Results of this study suggest that dietary enrichment with HSO increases the valuable nutrient content of the BSFL diet due to high levels of essential nutrients in both of them. This approach benefits both the environment and animal farming by reducing waste and producing high-quality insect-based animal feed.

## 1. Introduction

Sustaining environmental conservation is becoming an increasingly critical societal issue globally. Therefore, human cooperation in mitigating global environmental problems is imperative. According to projections by the Food and Agriculture Organization (FAO) of the United Nations, global food production needs to surge by approximately 70% by the year 2050 [1,2,3]. This significant increase underscores the growing pressure on agricultural systems to meet the demands of a burgeoning global population. Rising food demand has resulted in higher animal feed requirements, which currently constitute a substantial portion of production expenses in the livestock industry. Fish and soybean meal are primary protein sources for livestock, which also compete with human food production [4]. Currently, up to 15 million tons of seafood are used as ingredients in the animal feed industry, which is not only expensive but also costly to the environment [5]. Therefore, using black soldier fly larvae (BSFL) as an alternative nutrient source should mitigate the adverse environmental effects associated with animal husbandry practices, as well as facilitating decomposition of organic waste [6].

The black soldier fly (BSF), *Hermetia illucens* (Diptera; Stratiomyidae), originates from North America and exhibits several characteristics that distinguish it from common flies. Notably, the larval stages are incapable of flight and do not bite. Moreover, none of their life stages damage crops or serve as vectors for diseases that affect humans or animals [7]. Much research is being conducted to explore the various benefits of BSFL, such as: (1) serving as a cost-effective component in producing industrial animal feed; (2) being utilized in innovative waste management for plant and organic waste, thereby promoting environmental sustainability; and (3) functioning as a renewable energy source used in biofuel production. BSFL are nutritionally equivalent to fishmeal or soybean meal and are among the insect species approved for use in aquaculture feed by the European Union [8]. Besides aquaculture feed, the inclusion of BSFL in poultry feed reportedly improves the growth performance of broiler chickens, resulting in higher-quality meat [9].

Since BSFL can consume and decompose up to 80–90% of organic waste—up to five times faster than earthworms [10]—they play a crucial role in sustainable waste management. The decomposed organic waste is transformed into fertilizer, which improves soil fertility and supplies essential nutrients for plants. Additionally, BSFL are themselves rich in protein (42–56%) and fat (approximately 30%), as well as amino acids, vitamins, minerals, Omega-3, Omega-6, Omega-9, and chitin [11,12], which possess properties that inhibit the growth of various pathogens [13]. Given these advantages, BSFL are considered a promising solution for minimizing environmental impacts associated with animal husbandry. Their nutritional profile makes them suitable as feed for a wide range of livestock, including poultry, birds, fish, swine, rodents, reptiles, and amphibians [14]. Previous studies have found that BSFL are already used in the animal feed industry, in the United States for instance, for poultry, swine, trout, salmon, and shrimp [15,16].

The global BSFL market is projected to expand at a compound annual growth rate (CAGR) of 30.6%, increasing from US$108.7 million in 2019 to US$539.4 million by 2025. This market is primarily driven by the rising global demand for meat, aquaculture, and animal feed [17]. BSFL are possibly the most extensively studied insect larvae for their potential use as a protein source in animal feed. Previous studies reported that laying hens fed with BSFL, instead of soybean meal, significantly improved meat and egg quality in layers aged 24–45 weeks. Laying hens fed a diet containing 10% BSFL showed improvements in several parameters, such as increased egg and albumin weight, eggshell thickness, egg yolk color score, albumin height, and plasma calcium levels [18,19,20,21]. BSFL reared on different diets (e.g., vegetable waste, chicken feed, and kitchen waste) showed significant differences in nutrient contents such as protein, fat, and chitin. Therefore, enhancing the nutritional value of BSFL for use as animal feed is an important factor that should be taken into consideration.

Studying the utilization of agricultural by-products, especially hemp waste as a BSFL diet, has become increasingly popular in recent years. In this regard, hemp seed oil (HSO) is considered interesting due to its high content of polyunsaturated fatty acids (PUFAs) and various bioactive compounds. It is extracted from the hemp seeds of *Cannabis sativa* L., which contain significant amounts of protein, dietary fiber, phytochemicals, minerals, and unsaturated fatty acids (UFAs)—including omega-3 (linolenic acid), omega-6 (linoleic acid), and gamma-tocopherols [22,23]. A 3:1 ratio of omega-6 to omega-3 is desirable for health benefits [24]. Many studies have shown that HSO indeed has a variety of uses, both nutritionally and medicinally. In general, HSO has been used as both medicine and food in China for over 3000 years [25]. It is rich in phenolic compounds, which have shown anti-cancer effects in clinical trials. Moreover, some hemp-based products have been used for therapeutic treatment of cancer pain [26]. Furthermore, HSO also contains a significant amount of γ-linolenic acid, which has potential medical applications such as treatment of neurodermatitis and psoriasis [27,28]. In addition, some clinical trials have identified HSO as a functional food for feeding both humans and animal [29]. It is increasingly applied in the food, nutraceutical, cosmetic, and pharmaceutical industries, with global demand projected to rise from US$ 147.8 million in 2025 to US$ 855.7 million by 2035 (CAGR~19.2%) [30]. Previous research has demonstrated that the type of substrate provided to BSFL significantly influences their protein, lipid, and chitin content [31,32]. Given this background, the inclusion of HSO in the larval diet is expected to enhance the nutritional value of BSFL. Therefore, the aim of this study was to demonstrate the effects of adding HSO to BSFL diets on growth performances and the proximate and fatty acid profiles.

## 2. Materials and Methods

### 2.1. Black Soldier Fly Colony

The BSF colony was obtained from a laboratory strain maintained at the Prototype Black Soldier Fly Rearing Facility, Mae Hia Agricultural Research, Demonstration, and Training Center, Chiang Mai University, Thailand. Larvae were reared under ambient room conditions and provided with fresh soybean meal as the food source. After oviposition, eggs deposited in prepared oviposition devices were collected and incubated within 48 h. Upon reaching the first-instar stage, 500 larvae per replicate were selected for use in the experiment.

### 2.2. HSO Preparation

The hemp seeds used were a local variety grown in the Mae Sa Noi area in Mae Rim District, Pong Yaeng Subdistrict, Chiang Mai Province, Thailand. Before being used in the experiment, the harvested hemp seeds were subjected to a moisture reduction process and stored in a desiccant container until the content was below 12%. The dried seeds were placed in a cold-press screw oil expeller (FEA-100SS-M-H-TC-STAND, ENERGY FRIEND LTD., PART., Klong Luang, Thailand) and operated at a constant speed of 450 RPM, with the temperature controlled by an electric heater. The extracted oil was left in a sealed container overnight at a temperature of 32 ± 2 °C and then filtered to remove any remaining sediment. The filtered oil was then stored in a refrigerator at 4 °C for further testing.

### 2.3. Study of the Chemical Composition of HSO

HSO was analyzed using Gas Chromatography–Mass Spectrometry (GC-MS) with a Hewlett-Packard system (model 7890, Agilent Technologies, Santa Clara, CA, USA), following the method described by Suwannayod et al. [33]. The system setup included a split/splitless injector and an HP5 mass-selective detector (MSD) column with dimensions of 30 m × 0.25 mm ID × 0.25 µm film thickness connected to a 5975 mass spectrometer selective detector (Agilent Technologies, Santa Clara, CA, USA). The column temperature ranged from 50 °C to 250 °C at a ramp rate of 10 °C/min. The inlet temperature was maintained at 250 °C in splitless mode, with an injection volume of 0.5 µL. Helium was used as the carrier gas at a flow rate of 1.0 mL/min. Injections were made in split mode (250:1), and the total runtime was 24 min. The 5975 Network MSD (EI) was operated with scan parameters from 30 to 550 amu, with the mass selective (MS) Quadrupole temperature and MS source set at 150 °C and 230 °C, respectively. This analysis was conducted at the Science and Technology Service Center, Chiang Mai University (STSC-CMU).

### 2.4. Growth Performances

First instar BSF, aged 1 day post-hatching, were obtained from eggs originating from the same colony maintained at the Prototype BSF Rearing Facility, Mae Hia Agricultural Research, Demonstration, and Training Center, Chiang Mai University, Thailand. All of the eggs were collected within 48 h of oviposition to ensure uniform developmental stages. The base diet was formulated by mixing a 1:1 ratio of fresh soybean meal to rice bran (wet weight), with a moisture content of 70–80% relative humidity (RH). HSO was then incorporated at concentrations of 0.5%, 1%, 2%, 4%, and 6% (*w*/*w*) to create the treatment diets, while the control diet consisted of the base mixture without HSO supplementation. For each replicate, 500 first instar larvae were gently transferred into 10 g of the assigned diet using a No. 0 paintbrush moistened with saline and placed in a clear plastic box (14 cm × 25 cm × 8 cm), covered securely with taped fine mesh gauze to allow ventilation and prevent the larvae from escaping.

The larvae were maintained under ambient room temperature and RH conditions until pupation and fed daily until they ceased feeding and entered the pupal stage. The feeding dosage to the BSFL in each treatment group was 3 kg/week to ensure clarity and reproducibility. The growth and survival rate were recorded from the first instar stage until adult emergence, and a subset of pupae from each treatment group was collected to determine nutrient composition using standard proximate analysis methods (AOAC, 2005) [34]. Each treatment, including the control, was conducted with four independent replicates under identical rearing conditions.

### 2.5. Proximate Analysis

Each test and control group of the BSFL was examined to analyze nutrient content, including components such as crude protein, fat, ash, etc. The analysis was conducted by following the standards of the AOAC 2019 (Methods of Analysis for Nutrition Labeling 1993) [35] at the Central Laboratory (Thailand) Co., Ltd. in Chiang Mai, Thailand.

### 2.6. Fatty Acid Analysis

Each test and control group of the BSFL was thoroughly analyzed for fatty acid composition using the in-house method TE-CH-208, and following AOAC (2012) standards [36], at the Central Laboratory (Thailand) Co., Ltd. in Chiang Mai, Thailand.

### 2.7. Statistical Analysis

The survival rate (SR) was assessed to evaluate the impact of food by using the following equation:

$$\mathrm{SR}=\frac{\left(\;\mathrm{Number}\;\mathrm{of}\;\mathrm{surviving}\;\mathrm{larvae}\;\mathrm{at}\;\mathrm{the}\;\mathrm{end}\;\mathrm{of}\;\mathrm{the}\;\mathrm{experiment}\right)}{\left(\mathrm{Total}\;\mathrm{number}\;\mathrm{of}\;\mathrm{larvae}\;\mathrm{introduced}\;\mathrm{at}\;\mathrm{the}\;\mathrm{beginning}\;\mathrm{of}\;\mathrm{the}\;\mathrm{experiment}\right)}\;\times \;100\%$$

Data from various experiments were statistically analyzed using one-way analysis of variance (ANOVA) together with post hoc Tukey tests. The statistical analysis was conducted to determine differences in development time and SR (*p* < 0.05). All statistical analyses were performed using SPSS version 29.

## 3. Results

### 3.1. Chemical Composition of HSO

Table 1 shows the 24 chemical constituents identified in the HSO extract by using GC-MS analysis, which accounted for 87.44% of the total composition. The major bioactive components detected were 7-hexadecyne (19.39%), followed by γ-sitosterol (17.68%) and linolenic acid (17.34%). Notably, both omega-3 and omega-6 presented at 17.34% and 0.20%, respectively. Cannabidiol (CBD) was identified at 3.31%. The oil also contained several tocopherols, including γ-tocopherol (9.92%) and α-tocopherol (0.60%), indicating potential antioxidant properties. Additionally, various phytosterols such as campesterol (3.20%), β-stigmasterol (1.02%), and γ-sitosterol (17.68%) were abundant, further contributing to the bioactive profile of the oil. Other minor components such as 3,5-bis (P-dimethylaminostryl) (0.46%), dronabinol (1.88%), and oxirane (0.48%) were also identified. These diverse constituents reflect the complex chemical nature of HSO, with multiple compounds known for nutrition.

### 3.2. Growth Performances

The developmental performance of the BSFL, reared on supplemented diets with different concentrations of HSO, is summarized in Table 2. No significant differences (*p* > 0.05) were observed during larval development (L1-L4, L5, and L6 instars), prepupal, or pupal developmental times among all treatments compared with controls. Similarly, the larval and pupal SR remained high (above 85%) across all groups, without statistical differences (*p* > 0.05).

In contrast, larval weight exhibited a significant increase (*p* = 0.05) in HSO supplemented treatments compared to the control. The heaviest larvae were recorded at 0.5% HSO (0.155 ± 0.001 mg FM) and 1% HSO (0.154 ± 0.002 mg FM), whereas the control group had the lowest weight (0.141 ± 0.001 mg FM). However, larval width, length, and pupal weight did not differ significantly across treatments (*p* > 0.05). Adult longevity was unaffected by dietary HSO supplementation, with mean lifespan values ranging from 22.5 to 36.6 days and 19.8 to 33.75 days in males and females, respectively. Likewise, no significant differences were observed in pupal weight or adult survival parameters.

### 3.3. Proximate Composition and Fatty Acid Profile

**Figure 1:**
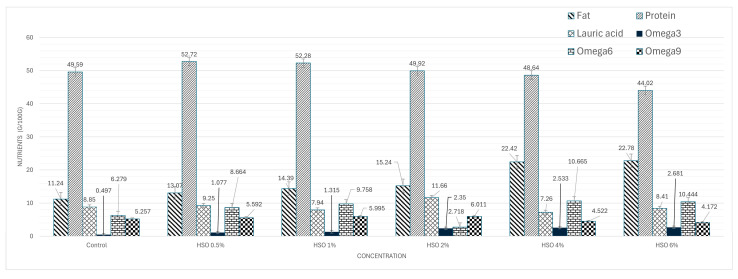
Nutritional response of BSFL to HSO concentrations.

This study showed that HSO included in the larval diet significantly altered the proximate composition of BSFL. The most notable effects were observed in fat and protein contents, which showed an inverse relationship across increasing HSO concentrations. Table 3 shows that fat content increased steadily from 11.24 g/100 g in the control group to a maximum of 22.78 g/100 g at 6% HSO (*p* < 0.05). The highest protein content was found in the HSO 0.5% group (52.72 g/100 g), followed by the HSO 1% group (52.28 g/100 g). A progressive decline in protein was observed at higher supplementation levels, with the lowest value recorded at 6% HSO (44.02 g/100 g). The control group value (49.59 g/100 g) has been retained as part of the comparative discussion (Table 3, Figure 1). The calculated energy content increased consistently with HSO supplementation, rising from 365.88 kcal/100 g in control group to a peak of 449.54 kcal/100 g at 4% HSO before slightly declining at 6% HSO (433.70 kcal/100 g) (Table 3).

The fatty acid profile of dried BSFL was significantly affected by dietary treatments, particularly in the levels of omega-3, omega-6, and omega-9 fatty acids (Table 3). Lauric acid content, which was 8.85 g/100 g in the control group, varied between 7.26 and 11.66 g/100 g when the HSO inclusion level increased to 4–6%. The concentration of omega-3 increased markedly (*p* < 0.05) across treatments, rising from 0.497 g/100 g in the control group to a maximum of 2.681 g/100 g at the highest supplementation level. In contrast, Omega-6 levels exhibited a non-linear pattern. They increased from 6.279 g/100 g in the control to 8.664 g/100 g at 0.5% HSO and 9.758 g/100 g at 1% HSO but markedly decreased to 2.718 g/100 g at 2% HSO before rising again to 10.665 and 10.444 g/100 g at 4% and 6% HSO, respectively (*p* < 0.05). For Omega-9, concentrations increased slightly from 5.257 g/100 g in the control to 5.592 and 5.995 g/100 g at 0.5% and 1% HSO, reached a maximum of 6.011 g/100 g at 2% HSO, and then declined to 4.522 and 4.172 g/100 g at 4% and 6% HSO, respectively (*p* < 0.05). (Table 3, Figure 1).

## 4. Discussion

### 4.1. Chemical Composition of HSO

GC-MS profiling of the HSO demonstrated a diverse range of bioactive constituents with potential nutritional and functional relevance. Notably, the oil was rich in UFAs, particularly linolenic (17.34%) and linoleic acid (0.20%), which are essentially involved in regulating metabolic functions, modulating immune responses, and maintaining membrane integrity in animals [29,37]. These compounds may influence larval development and physiological responses in insects when incorporated into the diet. Phytosterols were also prominently represented, with γ-sitosterol (17.68%), campesterol (3.20%), and β-stigmasterol (1.02%) being the most abundant. These sterols are known for their cholesterol-lowering, anti-inflammatory, and potential immunomodulatory properties [38]. Their occurrence in HSO suggests additional functional value, especially in the context of developing nutrient-enriched diets for insects such as the BSF. Cannabinoids including cannabidiol (CBD; 3.31%), cannabinol (2.06%), and cannabivarin (0.46%) were also detected. CBD has been associated with anti-inflammatory and neuroprotective activity [39]. Although these compounds are present in relatively small quantities compared to cannabis extracts, their bioactivity may contribute synergistically to larval health and stress tolerance. Antioxidants such as γ-tocopherol (9.92%) and α-tocopherol (0.60%) were identified, indicating that HSO possesses considerable oxidative stability. Tocopherols are lipid-soluble antioxidants that play a critical role in protecting cellular components against lipid peroxidation and oxidative stress [40], which are relevant factors in both larval development and adult longevity in holometabolous insects. Furthermore, the presence of hydrocarbons such as 7-hexadecyne (19.39%), nonacosane (1.13%), and cyclododecyne (1.38%) may have structural or antimicrobial functions, although their specific roles in insect physiology are not fully elucidated.

### 4.2. Growth Performance

These findings suggest that dietary supplementation with HSO at the tested concentrations did not interfere with larval instar transitions or the molting process in BSF, nor did it disrupt the formation of pupae. This indicates that the hormonal mechanisms governing metamorphosis remained functional despite alterations in dietary lipid composition [41,42]. Larval and pupal survival rates ranged from 84% to 99%, which falls within the expected range for BSF reared under controlled conditions. No statistically significant differences were observed among the treatment groups, further indicating that HSO inclusion did not compromise larval viability. Likewise, larval and pupal weights remained consistent across treatments, suggesting that the oil had no adverse effect on nutrient assimilation or biomass accumulation [9,43]. In addition, larval weight was significantly improved by HSO supplementation, particularly at 0.5–1%, where larvae showed higher body weights compared to the control. This enhancement may be attributed to the high nutritional value of HSO, which is rich in highly digestible protein and UFAs such as linoleic acid (omega-6) and α-linolenic acid (omega-3) [29,44]. These nutrients play essential roles in energy metabolism and cellular membrane synthesis. Previous studies have further demonstrated that dietary composition strongly influences the biochemical profile of BSFL, including protein and lipid content [14,45].

Regarding adult longevity, both male and female lifespans varied slightly among treatments, but did not differ significantly. Notably, females in the 4% HSO group exhibited longer lifespans (33.75 ± 7.76 days) compared to the control (26.4 ± 8.47 days), though the difference was not statistically significant. These results are in agreement with previous reports, indicating that BSF is highly tolerant to diverse feed substrates without compromising survival or growth performance [14,46]. However, further investigations had particular concerns about its potential impact on reproductive behavior or fecundity. These results are consistent with previous studies that reported BSF as having high dietary flexibility, especially in terms of lipid composition, provided that basic nutritional requirements are met [7,14,45]. Furthermore, the lack of significant differences in adult longevity between treatments supports earlier studies, which indicated that larval nutrition primarily affects body composition (e.g., protein and lipid profiles) but exerts limited influence on adult reproductive performance or lifespan [47,48]. Previous research demonstrated that increasing dietary energy density enhanced larval and adult body weight, fat reserves, and egg clutch quality in BSF by supplementing maize or fruit–vegetable substrates significantly. Their study confirmed a strong positive correlation between dietary energy, adipose tissue accumulation, and reproductive performance, emphasizing the quantitative role of energy supply in BSF development [41]. However, they also reported that the fatty acid profile of larval fat did not affect reproductive outcomes significantly. In comparison, this study with HSO supplementation highlights a different perspective. While growth and survival were not markedly altered, HSO improved the nutritional composition of larvae by enriching them with PUFAs and bioactive compounds. This suggests that HSO acts not only as an energy source but also as a functional additive that enhances the quality of BSF biomass. Together, these findings indicate that diet formulation plays a dual role: increasing energy density to promote reproductive efficiency and selecting lipid sources such as HSO to improve nutritional quality [41]. Moreover, this study aligns with previous ones, which demonstrated that substrate energy content, particularly from high-nutrient organic waste (e.g., university canteen leftovers), significantly enhanced larval growth and nutritional profile. While those studies primarily focused on growth performance and biochemical composition, the results of this study further elucidated the relationship between dietary energy density and body fat reserves, which are directly linked to reproductive performance. This distinction highlights the critical role of dietary formulation not only in larval biomass production, but also in long-term reproductive viability of BSF populations [49].

### 4.3. Macronutrient Composition

The proximate composition of dried BSFL was significantly influenced by dietary supplementation with HSO. The ash content also decreased significantly with increasing HSO, dropping from 9.34 g/100 g in the control group to 6.34 g/100 g at the highest supplementation level. This decline may suggest that a high-fat substrate dilutes mineral assimilation, potentially due to altered gut absorption dynamics or reduced mineral bioavailability in lipid-rich diets. This reduction may reflect a dilution effect due to the higher lipid content in the HSO-supplemented groups, which is consistent with diets rich in fat often reducing mineral concentration on a dry matter basis [45,50]. A similar reduction in mineral fractions with fat-enriched diets has been reported in *Tenebrio molitor* larvae, where dietary lipid supplementation lowered ash levels through changes in nutrient partitioning [51]. Fat content of the BSFL increased significantly with increasing HSO levels (*p* = 0.0001), reaching the highest value at 6% HSO (22.78 g/100 g), doubling that of the control group (11.24 g/100 g). This confirms that dietary lipid supplementation can be efficiently incorporated into larval biomass. The moisture content was significantly lower in most HSO treatments compared to the control, with the lowest values observed at 2% and 4% HSO (~8.25–8.31 g/100 g). Reduced moisture may be associated with higher lipid deposition in larval tissue, as lipid accumulation often inversely correlates with water content [7]. These findings indicate that dietary lipid enrichment promotes lipid accumulation in BSFL while simultaneously reducing protein deposition, which is consistent with nutrient allocation trade-offs reported in insect growth physiology.

Protein content ranged from 44.02 to 52.72 g/100 g and was significantly affected by treatments (*p* = 0.0005). Notably, larvae fed with 0.5–1% HSO exhibited the highest protein content (52.28–52.72 g/100 g), while higher supplementation levels (≥4%) tended to reduce protein proportion. This suggests that moderate HSO supplementation promotes protein deposition, whereas excessive lipid intake may displace protein accumulation in larval biomass. Similar findings revealed that dietary nutrient balance influences protein conversion efficiency in BSFL [48]. In terms of proximate composition, the percentage of protein increased at low–medium HSO inclusion (0.5–1%; 52.72–52.28 g/100 g) but declined at higher HSO (44.02 g/100 g at 6%). By contrast, lipid and energy rose markedly (11.24–22.78 g/100 g; 366–450 kcal/100 g), and moisture decreased in several HSO groups. These patterns are consistent with a protein-sparing effect and protein–fat trade-off within an energy-driven framework in insects; that is to say, dietary fat supplies energy that “spares” amino acids at low–moderate inclusion levels, whereas higher energy availability promotes fat deposition and lowers the protein proportion on a dry-matter basis—even if overall means do not reach statistical significance. This trend aligns with studies showing that increasing dietary energy/lipid stimulates body fat accumulation linked to adult performance in BSF, with diet formulation work demonstrating shifts in larval protein and lipid according to nutrient composition [41]. Energy content showed a steady increase with HSO supplementation (*p* = 0.0001), rising from 365.88 kcal/100 g in the control to a peak of 449.54 kcal/100 g at 4% HSO before slightly declining at 6% HSO (433.70 kcal/100 g). This pattern indicates that moderate lipid supplementation (2–4%) optimizes energy enrichment without compromising other nutritional traits, whereas excessive lipid inclusion may limit metabolic efficiency.

### 4.4. Fatty Acid Composition

The fatty acid composition of BSFL was markedly affected by dietary supplementation with HSO, indicating that larval lipid profiles are highly flexible and directly influenced by dietary lipid sources. The results for Lauric acid (C12:0) showed significant variation among treatments. The control group contained 8.85 g/100 g, while supplementation with HSO at 0.5% and 1% yielded 9.25 g/100 g and 7.94 g/100 g, respectively. Notably, the 2% HSO group exhibited the highest concentration at 11.66 g/100 g, which was significantly greater compared to the other treatments. In contrast, higher supplementation levels of 4% and 6% reduced the lauric acid content to 7.26 g/100 g and 8.41 g/100 g, respectively. These findings indicate that moderate HSO inclusion (2%) markedly enhanced lauric acid levels, whereas higher levels tended to suppress its accumulation. Similar findings reported that a diet supplemented with 1% flaxseed oil caused the BSFL n-6:n-3 ratio to decline because of the higher α-linolenic acid content, but no other n-3 PUFA [52]. In contrast, the inclusion of HSO markedly enriched UFAs, particularly PUFAs. The PUFAs increased consistently with higher supplementation, from 6.80 g/100 g in the control to 13.21–13.13 g/100 g at 4–6% HSO (*p* < 0.05). This reflects the high proportion of omega-3 and omega-6 fatty acids naturally present in HSO [29,44], which is efficiently incorporated into larval biomass. As a result, the total PUFA content increased substantially, while the total saturated fatty acid (SFA) proportion decreased with higher HSO levels. Notably, the omega-6 content rose more sharply than that of omega-3, leading to elevated absolute amounts of both classes of fatty acids but also altering the omega-6/omega-3 ratio. These results align with previous studies reporting that dietary substrates can be manipulated to enhance the PUFA profile of BSFL, thereby tailoring them for use in animal feed with improved nutritional quality [48,53]. Interestingly, monounsaturated fatty acids (MUFAs), particularly oleic acid (C18:1n9c), also showed a modest increase under HSO supplementation (5.24 g/100 g in control vs. 5.99 g/100 g at 1% HSO). This is consistent with the fact that HSO contains considerable amounts of MUFAs in addition to its PUFA richness [54].

This study showed that HSO supplementation at 0.5–6% had no adverse effects on BSFL growth or survival, while significantly enhancing their nutritional quality. Moderate inclusion improved larval weight and protein content, whereas higher levels increased lipid and energy deposition, reflecting a nutrient trade-off. When compared with a previous study, which tested multiple oils at 10% in Gainesville diets, both that and this study confirmed that dietary fatty acids are efficiently transferred into larval biomass, and that BSFL tolerate lipid-enriched diets. They highlighted flaxseed oil as the strongest enhancer of omega-3, with HSO providing intermediate but balanced enrichment. The findings in this study extend this knowledge by demonstrating that even low-to-medium HSO inclusion can effectively raise PUFA and energy content under practical feed conditions by using local substrates. Thus, HSO represents a sustainable feed additive that can improve BSFL nutritional profiles without compromising growth, while offering a valuable alternative for circular feed systems [55]. The fatty acid composition of BSFL is strongly influenced by the type of feed substrate used during rearing. In this study, clear differences were observed in the levels of individual fatty acids, highlighting the significant role of diet in shaping the biochemical composition of the larvae. These findings are consistent with several previous studies [55,56,57,58]. Consistent with this study’s hypothesis, HSO supplementation markedly shifted the BSFL omega-6:omega-3 ratio from a highly unbalanced ~12.6:1 in the controls toward a more favorable range (4–6% HSO ≈ 4.2–3.9:1), while 2% HSO yielded an n-3-dominant profile (~1.16:1). Together with the concurrent increases in absolute omega-3 and omega-6 contents, these results indicate efficient substrate-to- larva transfer and provide a practical lever for tailoring BSFL lipid quality toward target feed standards. The favorable amino acid balance in BSFL suggests its suitability as a protein complement in compound feed. However, nutrient profiles can vary depending on substrate, larval stage, and processing method, thus warranting further standardization and optimization for commercial-scale use.

### 4.5. Correlations Between HSO Dosage and Important Chemical Compositions as Well as Larvae Weight

The HSO dosage is defined as HSO concentration, HSO feed frequency, and duration of treatment. The HSO concentrations are as mentioned before, i.e., 0.5, 1, 2, 4, and 6%. The feeding schedules are on the first and eighth day of larvae age. The treatment duration is 15 days. Regarding the relationship between the HSO dosage and the resulting larvae weight at 15 days, each HSO concentration is normalized with the corresponding final larvae weight, i.e., the 15-day weight, which is the representative weight of the sample, to obtain the used dosage. This is based, however, on the assumption that the original weight of each larva is approximately equal. It should be noted that the survival rates in all cases are almost 100% (confer Table 2). The HSO concentration can be thus employed as the approximation of the used dosage as well. Pearson correlation analysis with the significance level of 0.05 is then applied to the computed dosage and each output, like resulting protein in dried BSF, to determine their respective correlation coefficients. Table 4 shows the correlation analysis results of important chemical compositions as well as 15-day larvae weight. From the results, the used dosage is positively correlated with omega-3 and negatively correlated with protein as well as omega-9, while there is no linear relationship with the other outputs. However, when considering Figure 1, there is a non-linear relationship between the used dosage and omega-6. This is also the case for the 15-day larvae weight.

## 5. Conclusions

This study demonstrated that dietary supplementation with HSO at concentrations of 0.5–6% had no significant impact on the developmental time, survival rate, biomass accumulation, or adult longevity of BSFL. These findings confirm the insect’s tolerance to moderate lipid variation in the diet, supporting its flexibility in feed formulation. Chemical profiling of HSO revealed a diverse array of bioactive compounds, including unsaturated fatty acids, phytosterols, cannabinoids, and tocopherols, which may contribute to functional properties if incorporated into insect feed. Moreover, proximate composition analysis of BSFL confirmed their nutritional value, highlighting high protein and fat contents, favorable amino acid profiles, and sufficient levels of essential minerals, making them a promising ingredient for sustainable animal feed. This finding supported the potential of integrating hemp by-products into BSF production systems without compromising insect performance. Future studies should investigate long-term effects, optimal dosages, and interactions with other dietary components to maximize nutritional benefits and system efficiency. Overall, these results highlight that HSO supplementation can strategically enhance the nutritional quality of BSFL, particularly in terms of fat and energy enrichment. The HSO improves the protein levels at the 0.5–1.0% concentration, with negative correlations at higher concentrations. This nutritional modulation supports the use of HSO as a feed additive to tailor BSF composition for applications in livestock and aquaculture feed.

## Figures and Tables

**Table 1:** Chemical composition of compounds identified in HSO (*C. sativa*) using GC-MS analysis.

No.	Chemical Constituents	RT (min)	Percentage Composition (%)
1	2-heptenal	6.421	0.47
2	2,4-decadienal	16.240	0.37
3	linoleic acid	34.110	0.20
4	δ.1-tetrahydrocannabidivarol	37.206	0.81
5	cannabivarin	38.178	0.46
6	cannabidiol	38.951	3.31
7	7-hexadecyne	39.798	19.39
8	linolenic acid	39.895	17.34
9	cyclododecyne	40.055	1.38
10	dronabinol	40.547	1.88
11	9,12-Octadecadienoic acid	41.056	0.29
12	cannabinol	41.514	2.06
13	eicosane	43.368	0.32
14	skyvalen	44.919	1.96
15	oxirane	45.285	0.48
16	nonacosane	46.166	1.13
17	3,5-bis (P-dimethylaminostryl)	46.612	0.46
18	γ-tocopherol	48.014	9.92
19	α-tocopherol	49.016	0.60
20	campesterol	50.183	3.20
21	β-stigmasterol	50.526	1.02
22	γ-sitosterol	51.367	17.68
23	4-nitrophthalhydrazide	52.065	0.80
24	cycloartenol	52.552	1.91
Total			87.44

**Table 2:** BSF developmental growth with different HSO concentrations compared to the control.

Specification	Treatments (Mean ± SE)						
	Control	HSO 0.5%	HSO 1%	HSO 2%	HSO 4%	HSO 6%	*p*-Value
Larval developmental time (L1–L4 days)	14.5 ± 0.55	14.33 ± 0.82	14.5 ± 1.22	15 ± 1.10	14.5 ± 1.05	14.5 ± 0.05	0.856
Larval developmental time (L5 days)	15.5 ± 0.55	15.67 ± 1.21	15.5 ± 1.22	16.17 ± 1.17	15.67 ± 1.21	16.10 ± 1.15	0.829
Prepupal developmental time (L6 days)	17.5 ± 0.55	17.5 ± 1.52	16.67 ± 1.03	17.17 ± 1.17	17.5 ± 1.52	17.55 ± 1.40	0.704
Pupal developmental time (days)	20.33 ± 1.51	20 ± 2.45	19.83 ± 1.17	19.67 ± 1.21	19.5 ± 1.22	19.80 ± 1.10	0.417
Larval survival rate (%)	98.8 ± 2.67	98.67 ± 2.44	98.67 ± 1.11	98.73 ± 1.55	99.47 ± 1.56	99.13 ± 2.22	0.594
Pupal survival rate (%)	91.67 ± 0.03	88.67 ± 2.03	84.00 ± 1.21	86.67 ± 1.55	86.33 ± 1.15	86.67 ± 1.40	0.864
Larval weight (mg FM)	0.141 ± 0.001 ^a^ *	0.155 ± 0.001 ^c^	0.154 ± 0.002 ^c^	0.151 ± 0.0001 ^b^	0.151 ± 0.0006 ^b^	0.153 ± 0.0001 ^b^	0.001
Larval Width (cm)	0.259 ± 0.18	0.259 ± 0.17	0.280 ± 0.17	0.469 ± 0.03	0.509 ± 0.03	0.500 ± 0.03	0.085
Larval Length (cm)	1.449 ± 0.15	1.482 ± 0.15	1.502 ± 0.15	1.571 ± 0.12	1.731 ± 0.07	1.690 ± 0.04	0.955
Pupal weight (mg FM)	0.195 ± 0.04	0.194 ± 0.04	0.207 ± 0.03	0.197 ± 0.04	0.204 ± 0.04	0.204 ± 0.04	0.854
Adult longevity male (days)	36 ± 6.22	36.6 ± 9.61	23.5 ± 0.5	22.5 ± 2.51	34 ± 6.68	34 ± 3.25	0.594
Adult longevity female (days)	26.4 ± 8.47	19.8 ± 18.77	23.5 ± 0.71	28 ± 2.00	33.75 ± 7.76	32.50 ± 2.25	0.417

* Means within the same row followed by the same letter are not significantly different (*p* < 0.05, one-way ANOVA).

**Table 3:** Chemical composition of analyzed macronutrients, minerals and selected amino acids of dried BSFL.

Nutrients (g/100 g)	Control	HSO 0.5%	HSO 1%	HSO 2%	HSO 4%	HSO 6%	*p*-Value
Ash	9.34 ^a^	9.24 ^a^	9.27 ^a^	8.71 ^b^	7.33 ^c^	6.34 ^d^	0.0003
Fat	11.24 ^e^	13.07 ^d^	14.39 ^c^	15.24 ^b^	22.42 ^a^	22.78 ^a^	0.0001
Moisture	13.24 ^a^	9.22 ^b^	8.53 ^b^	8.25 ^b^	8.31 ^b^	13.71 ^a^	0.0021
Protein	49.59 ^b^	52.72 ^a^	52.28 ^a^	49.92 ^b^	48.64 ^c^	44.02 ^d^	0.0005
Energy (kcal/100 g)	365.88 ^e^	391.51 ^d^	400.75 ^c^	408.36 ^b^	449.54 ^a^	433.70 ^a^	0.0001
Fatty acid composition							
Caproic acid (C6:0)	-	-	-	0.06	-	-	
Caprylic acid (C8:0)	-	-	-	0.05	0.01	-	
Capric acid (C10:0)	0.30 ^a^	0.32 ^a^	0.29 ^a^	0.04 ^b^	0.27 ^a^	0.28 ^a^	
Undecanoic acid (C11:0)	-	-	-	0.02	-	-	
Lauric acid (C12:0)	8.85 ^b^	9.25 ^b^	7.94 ^c^	11.66 ^a^	7.26 ^c^	8.41 ^bc^	
Tridecanoic acid (C13:0)	0.01	0.01	0.01	0.02	-	-	
Myristic acid (C14:0)	1.41 ^a^	1.45 ^a^	1.25 ^b^	1.92 ^a^	1.16 ^b^	1.33 ^b^	
Pentadecanoic acid (C15:0)	0.07 ^a^	0.07 ^a^	0.06 ^ab^	0.09 ^a^	0.04 ^b^	0.04 ^b^	
Palmitic acid (C16:0)	3.88 ^c^	3.91 ^c^	4.10 ^b^	5.79 ^a^	3.93 ^c^	3.87 ^c^	
Heptadecanoic acid (C17:0)	0.88 ^a^	0.07 ^b^	0.06 ^b^	0.09 ^b^	0.05 ^b^	0.05 ^b^	
Stearic acid (C18:0)	0.66 ^c^	0.68 ^c^	0.69 ^c^	1.09 ^a^	0.79 ^b^	0.82 ^b^	
Arachidic acid (C20:0)	0.03 ^d^	0.03 ^d^	0.04 ^c^	0.07 ^a^	0.05 ^b^	0.06 ^a^	
Heneicosanoic acid (C21:0)	0.01	-	-	0.02	-	-	
Behenic acid (C22:0)	0.02	0.02	0.02	0.06	0.03	-	
Tricosanoic acid (C23:0)	0.01 ^b^	0.01 ^b^	0.01 ^b^	0.06 ^a^	0.01 ^b^	0.01 ^b^	
Lignoceric acid (C24:0)	-	-	-	0.01	-	-	
Saturated fat (g/100 g)	15.38 ^c^	15.86 ^b^	14.51 ^e^	21.42 ^a^	13.64 ^f^	14.92 ^d^	
Myristoleic acid (C14:1)	0.01	-	-	-	-	-	
cis-10-Pentadecenoic acid (C15:1n10)	-	-	-	-	-	-	
Plamitoleic acid (C16:1n7)	0.67 ^a^	0.63 ^b^	0.61 ^c^	0.56 ^d^	0.46 ^e^	0.30 ^f^	
cis-10-Heptadecenoic acid (C17:1n10)	0.03 ^a^	0.03 ^a^	0.03 ^a^	0.03 ^a^	0.02 ^a^	0.01 ^b^	
Trans-9-Elaidic acid (C18:1n9t)	0.07 ^a^	0.05 ^b^	0.05 ^b^	0.06 ^a^	0.02 ^c^	0.02 ^c^	
cis-9-Oleic acid (C18:1n9c)	5.24 ^d^	5.58 ^c^	5.99 ^a^	5.95 ^b^	4.51 ^e^	4.16 ^f^	
cis-11-Eicosenoic acid (C20:1n11)	0.01 ^b^	0.02 ^a^	0.02 ^a^	0.03 ^a^	0.03 ^a^	0.03 ^a^	
Nervonic acid (C24:1n9)	0.01	0.01	-	0.06	0.01	0.01	
Monounsaturated fatty acid	6.05 ^d^	6.33 ^c^	6.71 ^a^	6.69 ^b^	5.05 ^e^	4.54 ^f^	
cis-9,12-Linoleic acid (C18:2n6)	6.26 ^e^	8.65 ^d^	9.74 ^c^	2.69 ^f^	10.64 ^a^	10.43 ^b^	
gamma-Linolenic acid (C18:3n6)	-	-	0.01	0.02	0.01	0.01	
alpha-Linolenic acid (C18:3n3)	0.49 ^e^	1.08 ^d^	1.32 ^c^	0.22 ^f^	2.52 ^b^	2.68 ^a^	
cis-11, 14-Eicosadienoic acid (C20:2)	0.02	0.01	0.02	0.04	0.01	-	
cis-8, 11, 14-Eicosatrienoic acid (C20:3n6)	-	-	-	0.02	-	-	
cis-5, 8, 11, 14, 17-Eicosapentaenoic acid (C20:5n3)	-	-	-	0.01	-	-	
Polyunsaturated Fatty acid	6.80 ^e^	9.75 ^d^	11.09 ^c^	2.99	13.21 ^a^	13.13 ^b^	
Unsaturated fat	12.85 ^e^	16.09 ^d^	17.80 ^b^	9.68 ^f^	18.26 ^a^	17.67 ^c^	
Tran fat	0.07 ^a^	0.05 ^b^	0.05 ^b^	0.06 ^a^	0.02 ^c^	0.02 ^c^	
Omega-3	0.497 ^f^	1.077 ^e^	1.315 ^d^	2.350 ^c^	2.533 ^b^	2.681 ^a^	0.0001
Omega-6	6.279 ^e^	8.664 ^d^	9.758 ^c^	2.718 ^f^	10.665 ^a^	10.444 ^b^	0.0001
Omega-9	5.257 ^d^	5.592 ^c^	5.995 ^b^	6.011 ^a^	4.522 ^e^	4.172 ^f^	0.0001

Chemical composition (g/100 g) within the same row followed by the same letter is not significantly different (*p* < 0.05, one-way ANOVA).

**Table 4:** Correlation analysis between HSO dosage and important chemical compositions as well as 15-day larvae weight.

Nutrients	Correlation Coefficient	*p*-Value
Protein	−0.98	0.0005 ***
Lauric	−0.308	0.326
Omega-3	0.873	0.029 ***
Omega-6	0.357	0.299
Omega-9	−0.9	0.018 ***
Weight	−0.447	0.248

*** significance level (*p* < 0.05).

## Data Availability

The original contributions presented in this study are included in the article. Further inquiries can be directed to the corresponding author.
